# Supplementary material for: citrOgen: a synthesis-free polysaccharide and protein antigen-presentation to antibody-induction platform
Source: Nat Commun. 2025 Oct 6;16:8886. doi: 10.1038/s41467-025-63922-0 (PMC12501221; doi:10.1038/s41467-025-63922-0)
Supplement: Supplementary file 1 — Supplementary Information [file 41467_2025_63922_MOESM1_ESM.pdf]

Supplementary Information

citrOgen: a synthesis-free polysaccharide and protein antigen-presentation to antibody-induction platform

Wong and Sanchez-Garrido et al.

SUPPLEMENTARY FIGURE 1.

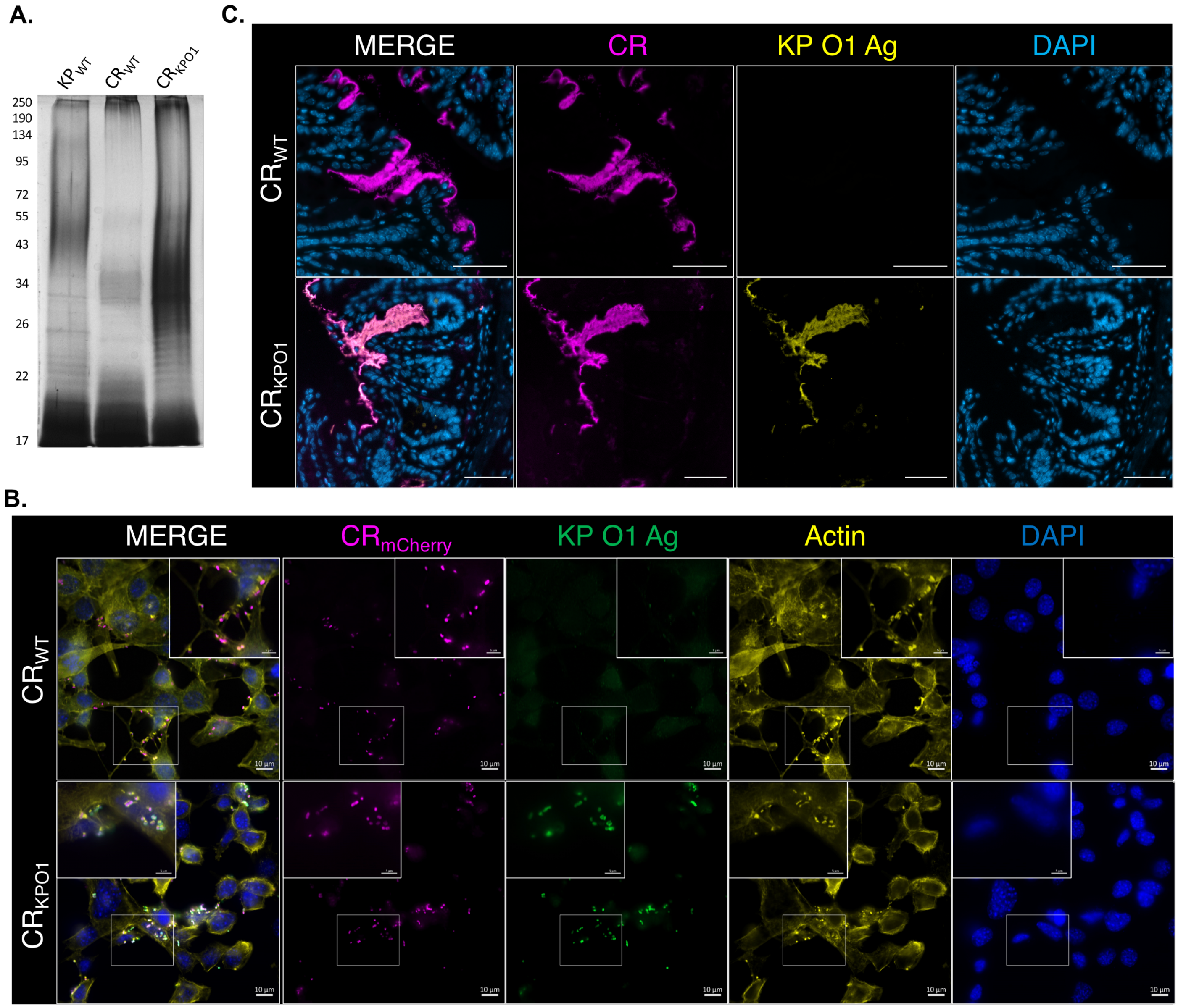

Supplementary Figure 1 (related to Fig. 1). CR expressing a heterologous KP O-antigen (KPO1) triggers actin polymerisation *in vitro* but exhibits an *in vivo* colonisation defect.

A. Silver stain SDS-PAGE gel of crude LPS samples from *K pneumoniae* wild-type (KP_{WT}), *C. rodentium* wild-type (CR_{WT}) and CR expressing KPO1 (CR_{KPO1}). Molecular weights (in kDa) are shown on the left.

B. Representative images of immunostaining of Swiss-3T3 cells infected with the indicated CR strains expressing pUltra-mCherry. CR_{KPO1} forms actin-rich pedestals (phalloidin F-actin stain) and is immunoreactive to an anti-KP O1 Ag monoclonal antibody (C13 mAb); nuclei were stained with 4',6-diamidino-2-phenylindole (DAPI). Scale bar = 10 µm.

C. Zoomed images of colonic sections at 8 dpi and stained for CR and KP O1 Ag; DAPI staining used as a structural marker. CR_{WT} has no detectable KP O1 Ag expression while CR_{KPO1} presents clear KP O1 Ag expression. Scale bar = 50 µm.

Images are representative of 2 (A and B) and 3 (C) biologically independent repeats.

SUPPLEMENTARY FIGURE 2.

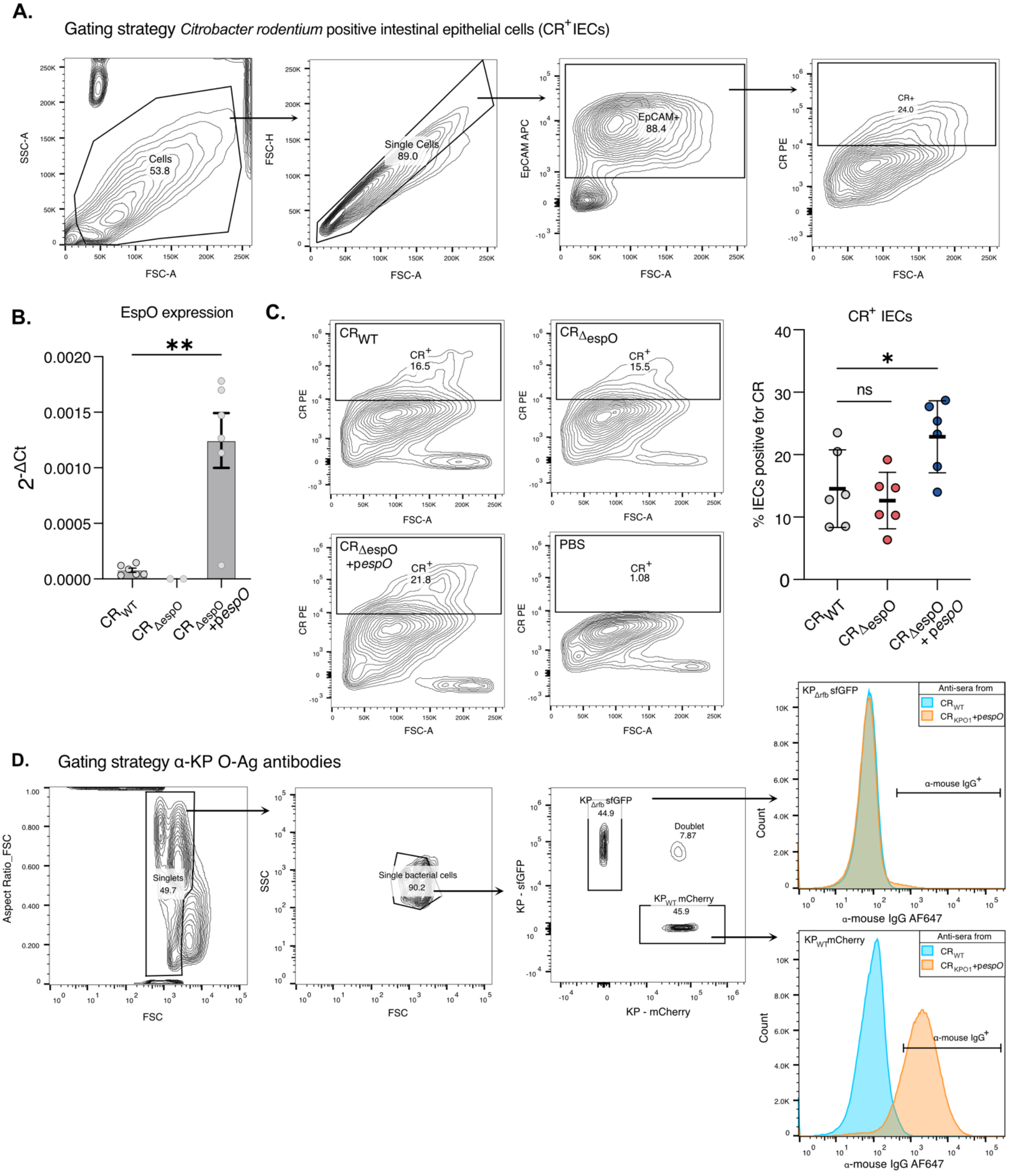

Supplementary Figure 2 (related to Fig. 2). Overexpression of EspO in CR_{KPO1} increases attachment to IECs and induces specific anti-KP O1 Ag IgG responses.

A. Gating strategy for the measurement of CR-positive (CR^+) IECs (defined as EpCAM-positive).

B. *espO* expression was significantly higher in the presence p*espO* as determined by qRT-PCR in bacterial cultures grown statically in DMEM (T3SS-inducing conditions); $CR_{\Delta espO}$ was used as a negative control. Graph shows mean±SEM from 6 biological repeats; statistical significance was determined by paired t-test between CR_{WT} and $CR_{\Delta espO}$pespO; ** p<0.01.

C. The proportion of CR^+ IECs isolated from the distal colon is higher in mice infected with CR over-expressing *espO* from pACYC-*espO* ($CR_{\Delta espO}$+p*espO*); representative flow cytometry plots are shown on the left. Graph shows mean±SEM from 6 biological repeats; statistical significance was determined by repeated measured one-way ANOVA corrected with Dunnett's multiple comparisons test; * $p<0.05$. Source data are provided in the Source Data file.

D. Gating strategy used to test the binding specificity of citrOgen-derived antibodies against KP O1-Ag using KP_{WT} expressing mCherry and $KP_{\Delta rfb}$ expressing sfGFP. Representative histograms of the two populations are shown on the right, where the anti-sera from CR_{KPO1}-infected mice is shown to bind KP_{WT} but not $KP_{\Delta rfb}$, antisera from CR_{WT}-infected mice were used as a negative control.

SUPPLEMENTARY FIGURE 3.

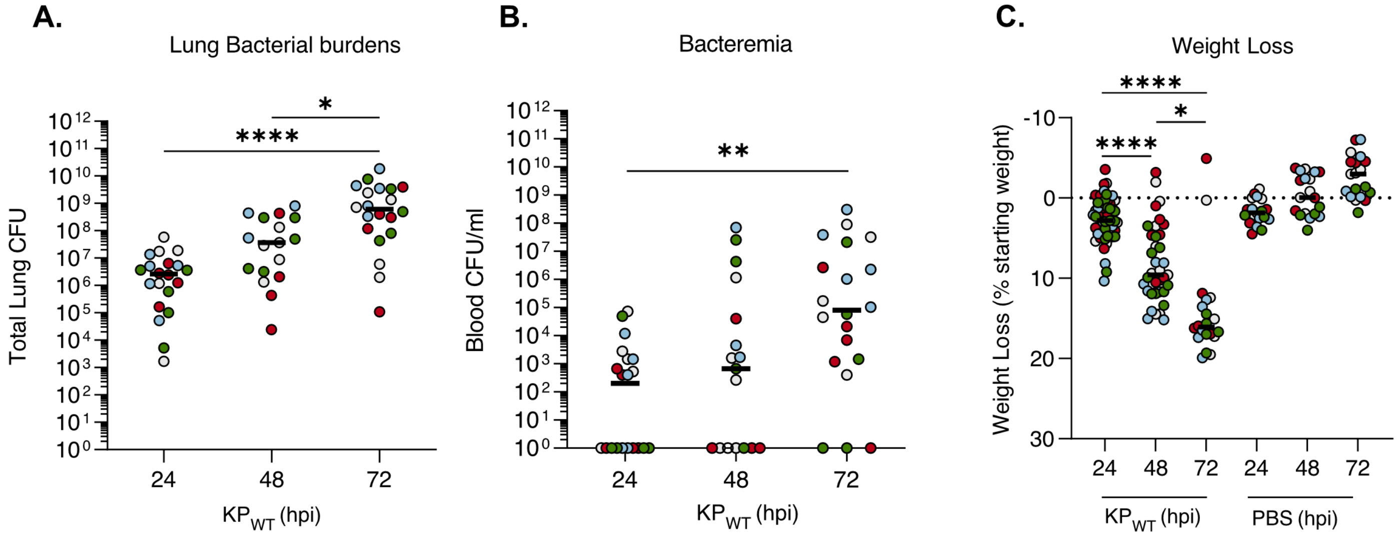

Supplementary Figure 3. KP$_{WT}$ lung infection (500CFU) of CD-1 mice results in consistent bacterial replication and disease progression.

A. KP$_{WT}$ burden in the lungs undergoes a stepwise daily increase, reaching significance by 72 hpi compared to 24 and 48 hpi.

B. The proportion of bacteraemic mice increases as the infection progresses; by 72 hpi 85% of the infected mice are bacteraemic.

C. KP$_{WT}$ infection induces significant temporal weight loss; no weight loss was seen in the PBS mock-infected mice.

A-C. Graphs are representative of 4 biological repeats where each repeat is represented by a different colour. Statistical significance was determined by non-parametric Kruskal-Wallis test corrected with Dunn's multiple comparisons test; in (C) significance was tested between the timepoints within a treatment group. * $p<0.05$; ** $p<0.01$; **** $p<0.0001$. Source data are provided in the Source Data file.

SUPPLEMENTARY FIGURE 4.

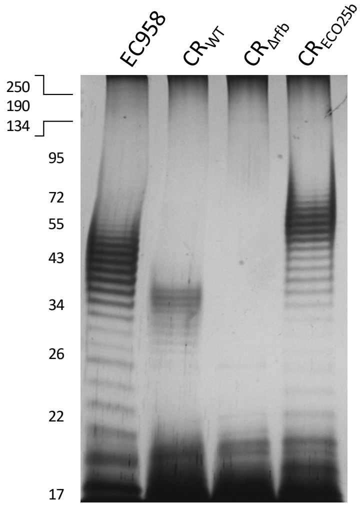

Supplementary Figure 4 (related to Fig. 4). The citrOgen platform can be used to express O-antigens exported through the more common *wzy* pathway

Silver stain SDS-PAGE gel of crude LPS samples from *E.coli* ST131 expressing O25b (EC958), *C. rodentium* wild-type (CR$_{WT}$), CR lacking O-antigen expression (CR$_{\Delta rfb}$) and CR expressing ST131 O25b (CR$_{ECO25b}$). Molecular weights (in kDa) are shown on the left.

SUPPLEMENTARY FIGURE 5.

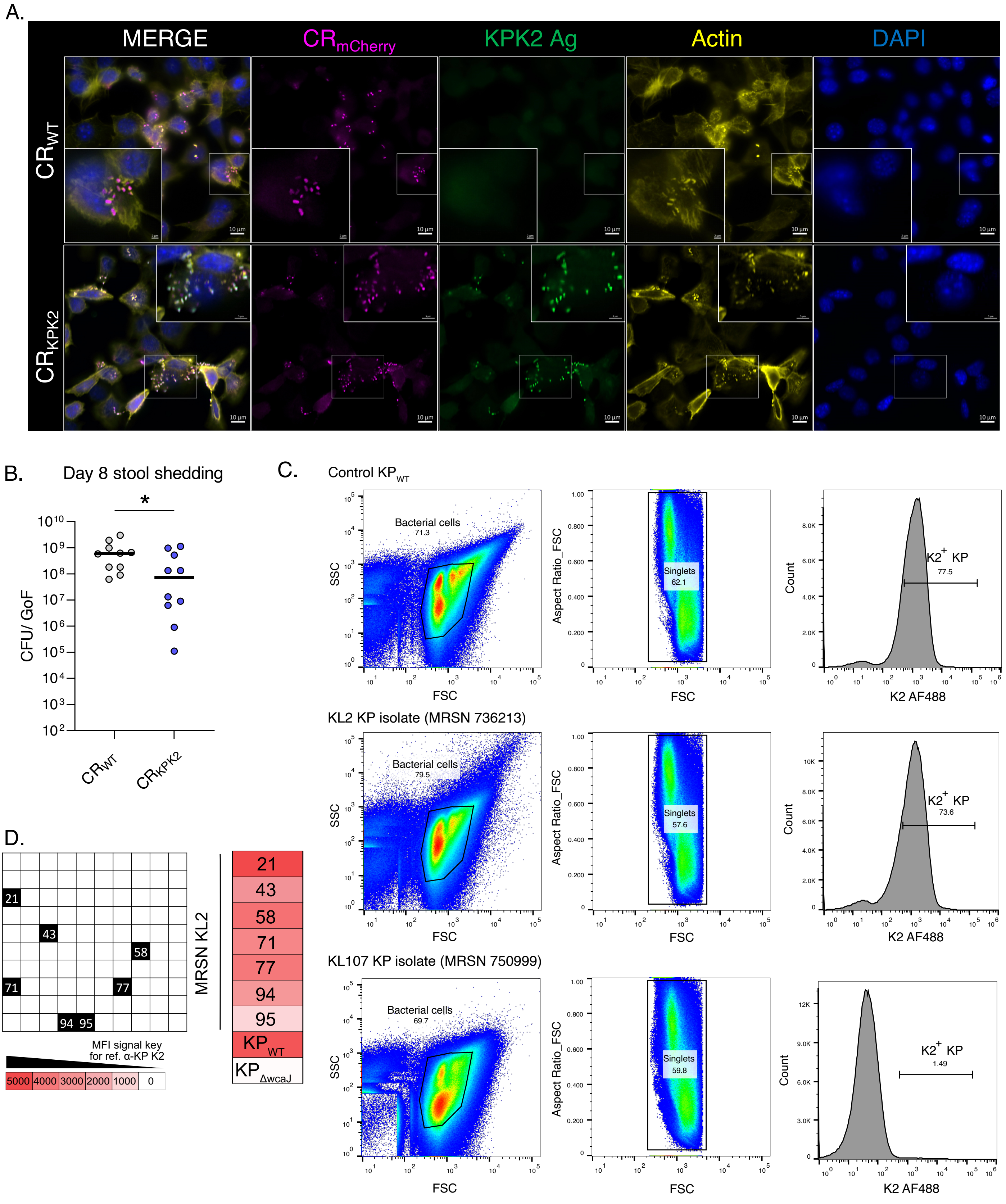

Supplementary Figure 5 (related to Fig. 5). Anti-sera from CR_{KPK2}-infected mice can be used for KP capsule serotyping.

A. Representative images of Swiss-3T3 cells infected with the indicated CR strains expressing pUltra-mCherry and stained with anti-KP K2 antibody (SSI), phalloidin (F-actin stain) and DAPI (nucleic acid stain). CR_{KPK2} forms actin-rich pedestals while being immunoreactive to anti K2 KP antibodies. Scale bar = 10 µm. Images are representative of 2 biologically independent replicates.

B. At 8 dpi following oral gavage, faecal CFUs are significantly lower in CR_{KPK2} compared with CR_{WT} . Graph shows median of n=10 mice per group, 2 biological repeats. Statistical significance was determined using a lognormal Welch's two-sided, unpaired t test. *, $P<0.05$. Faecal CFUs for CR_{WT} are as in Supplementary Figure 6C. GoF, gram of faeces. Source data are provided in the Source Data file.

C. Representative flow cytometry dot plots obtained after staining the MRSN collection of 100 KP isolates with citrOgen-derived anti-KP K2 antibodies; KP_{WT} is used as a positive control. MRSN 736613 is shown to be a KL2-expressing isolate while MRSN 750999 is negative for KL2-staining, corroborating with the genomic analysis of these isolates predicted to be KL2 and KL107 respectively.

D. The reference anti-KP K2 antibody (SSI) was used to validate the results obtained for the KL2 strains from the collection, highlighted as numbered black squares in the schematic to the left, based on Fig 5E. KP_{WT} and the capsule-deficient $KP_{\Delta wcaJ}$ were used as controls. The boxes are coloured according to the strength of binding by the anti-KP K2 antibody, which was determined using the median fluorescence intensity obtained by flow cytometry analysis.

SUPPLEMENTARY FIGURE 6.

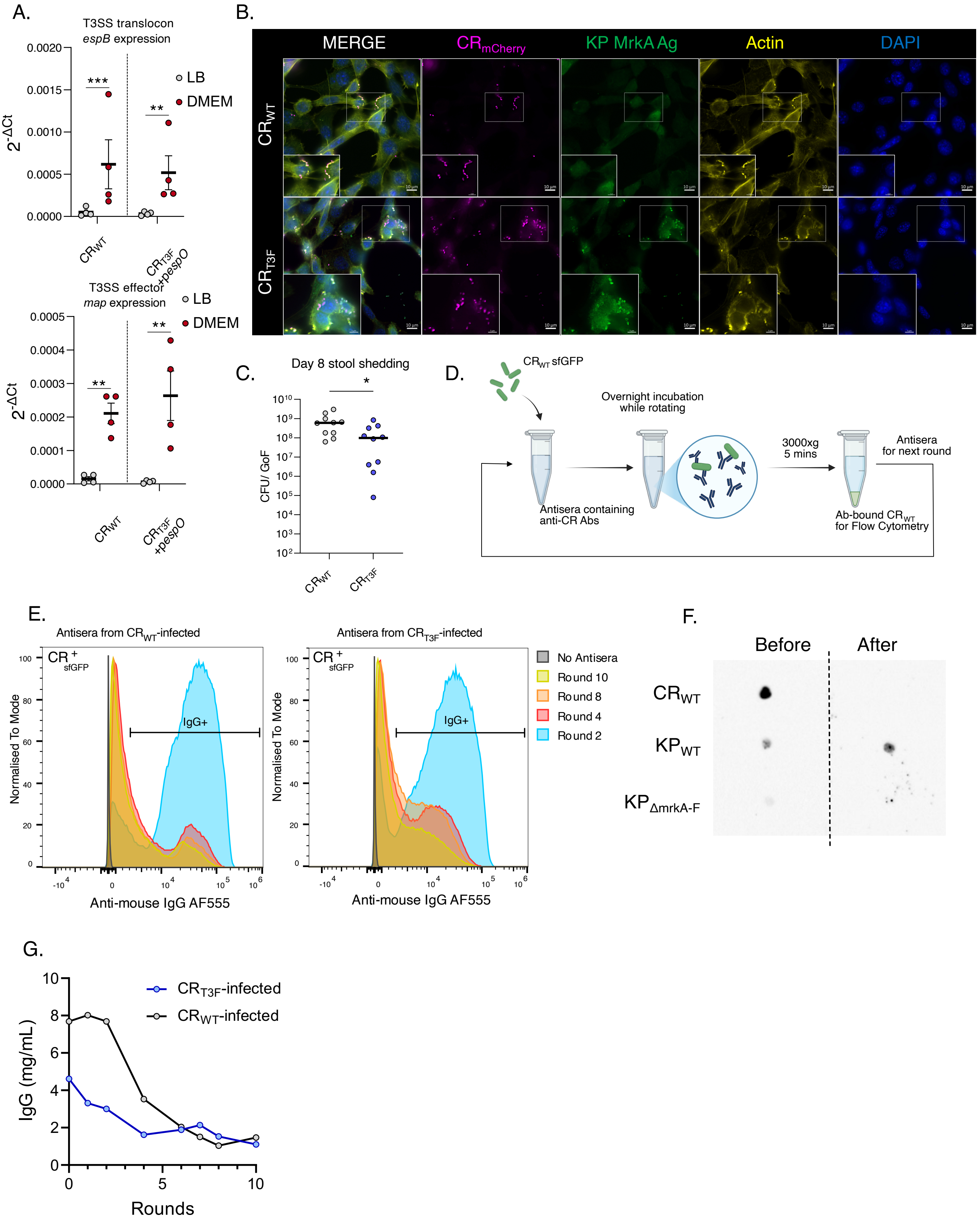

Supplementary Figure 6 (related to Fig. 6). A complex protein Ag, KP T3F, expressed by citrOgen under the *map* promoter, is used to generate functional anti-KP T3F Abs.

A. qRT-PCR was used to quantify the expression levels of the structural T3SS gene *espB* (top) and the T3SS effector *map* (bottom) in bacterial cultures of CR_{WT} and CR_{T3F} grown statically under T3SS suppressing (LB) and inducing (DMEM)

conditions. T3SS-inducing conditions similarly induce *espB* and *map* transcription in CR_{WT} and CR_{T3F}. Results are from 4 biological replicates; graphs show central tendency mean± SEM. Statistical significance was determined by ratio-paired two-sided t-tests comparing expression in LB and DMEM; ** $p<0.01$; *** $p<0.001$.

B. Representative images if Swiss 3T3 mouse fibroblasts infected with the indicated mCherry-tagged CR strains and immunostained with an Ab against MrkA, phalloidin (F-actin) and DAPI (nuclei). CR_{T3F} forms actin-rich pedestals to the same extent as CR_{WT} while expressing type 3 fimbriae (T3F), which also increased attachment to the glass coverslips. Scale bar=10 µm. Images are representative of 2 biological replicates.

C. At 8 dpi following oral gavage, faecal CFUs are significantly lower in CR_{T3F} compared with CR_{WT} . Graph shows median of n=10 mice per group from 2 biological repeats. Statistical significance was determined using a lognormal Welch's two-sided, unpaired t test. *, $P<0.05$. Faecal CFUs for CR_{WT} are as in Supplementary Figure 5B. GoF, gram of faeces.

D. Schematic representing the protocol used to pre-adsorb citrOgen-derived antisera using PFA-fixed CR expressing sfGFP (CR_{WT}sfGFP) to remove CR_{WT} Abs. Created using BioRender. Frankel, G. (2025) https://BioRender.com/m6egure

E. Flow cytometry histograms of the antisera obtained from CR_{WT}- (left) and CR_{T3F}-infected (right) mice. which has undergone the indicated number of rounds of pre-adsorption against CR_{WT}. While by round 2 of pre-adsorption most of the antisera still bound CR_{WT}, by round 10 there is negligible binding to CR_{WT}.

F. Dot-blot performed against the indicated bacterial strains with antisera from CR_{T3F}-infected mice before and after 10 rounds of pre-adsorption normalised to the total IgG content; following pre-adsorption the antisera only bind KP_{WT}.

G. Levels of total IgG in the antisera from CR_{WT} and CR_{T3F}-infected mice assessed by ELISA, measured before, during and after the pre-adsorption process.

Source data are provided in the Source Data file.
