# Supplementary material for: citrOgen: a synthesis-free polysaccharide and protein antigen-presentation to antibody-induction platform
Source: Nat Commun. 2025 Oct 6;16:8886. doi: 10.1038/s41467-025-63922-0 (PMC12501221; doi:10.1038/s41467-025-63922-0)
Supplement: Supplementary file 2 — Description of Additional Supplementary Information [file 41467_2025_63922_MOESM2_ESM.pdf]

Description of Additional Supplementary Files

File Name: Supplementary Data 1
Description: Strains used in this study

File Name: Supplementary Data 2
Description: Mutagenesis vectors

File Name: Supplementary Data 3
Description: Antibodies used in this study

File Name: Supplementary Data 4
Description: RT-qPCR primers

File Name: Supplementary Data 5
Description: In vivo outcome variables
