# Supplementary material for: citrOgen: a synthesis-free polysaccharide and protein antigen-presentation to antibody-induction platform
Source: Nat Commun. 2025 Oct 6;16:8886. doi: 10.1038/s41467-025-63922-0 (PMC12501221; doi:10.1038/s41467-025-63922-0)
Supplement: Supplementary file 4 — Reporting Summary [file 41467_2025_63922_MOESM4_ESM.pdf]

nature portfolio

Corresponding author(s): DAPR NCOMMS-25-18013B

Last updated by author(s): Aug 12, 2025

Reporting Summary

Nature Portfolio wishes to improve the reproducibility of the work that we publish. This form provides structure for consistency and transparency in reporting. For further information on Nature Portfolio policies, see our Editorial Policies and the Editorial Policy Checklist.

Statistics

For all statistical analyses, confirm that the following items are present in the figure legend, table legend, main text, or Methods section.

n/a	Confirmed	
☐	☒	The exact sample size (*n*) for each experimental group/condition, given as a discrete number and unit of measurement
☐	☒	A statement on whether measurements were taken from distinct samples or whether the same sample was measured repeatedly
☐	☒	The statistical test(s) used AND whether they are one- or two-sided<br>*Only common tests should be described solely by name; describe more complex techniques in the Methods section.*
☒	☐	A description of all covariates tested
☐	☒	A description of any assumptions or corrections, such as tests of normality and adjustment for multiple comparisons
☐	☒	A full description of the statistical parameters including central tendency (e.g. means) or other basic estimates (e.g. regression coefficient) AND variation (e.g. standard deviation) or associated estimates of uncertainty (e.g. confidence intervals)
☒	☐	For null hypothesis testing, the test statistic (e.g. *F*, *t*, *r*) with confidence intervals, effect sizes, degrees of freedom and *P* value noted<br>*Give P values as exact values whenever suitable.*
☒	☐	For Bayesian analysis, information on the choice of priors and Markov chain Monte Carlo settings
☒	☐	For hierarchical and complex designs, identification of the appropriate level for tests and full reporting of outcomes
☒	☐	Estimates of effect sizes (e.g. Cohen's *d*, Pearson's *r*), indicating how they were calculated

Our web collection on statistics for biologists contains articles on many of the points above.

Software and code

Policy information about availability of computer code

Data collection	Cytek AMNIS CellStream acquisition software 1.3.384, Image Lab 6.1 , ZEN3.1 (blue edition), Quantstudio 1 System, BD FACSDiva v9.0 Software, BMG Labtech Omega Control version 5.11 R3, Abaxis HM5 version 2.5.
Data analysis	FlowJo v10.10.0, GraphPad Prism version 10.4.1 for Windows and version 10.4.0 for Mac, SerotypeFinder 2.0, QuantStudio Design & Analysis Software v1.5.2, Gpower 3.1, Benchling (cloud-based web app), BMG Labtech Mars Omega data analysis version 3.31.

For manuscripts utilizing custom algorithms or software that are central to the research but not yet described in published literature, software must be made available to editors and reviewers. We strongly encourage code deposition in a community repository (e.g. GitHub). See the Nature Portfolio guidelines for submitting code & software for further information.

Data

Policy information about availability of data

All manuscripts must include a data availability statement. This statement should provide the following information, where applicable:

- Accession codes, unique identifiers, or web links for publicly available datasets
- A description of any restrictions on data availability
- For clinical datasets or third party data, please ensure that the statement adheres to our policy

All plasmid vector sequences are publicly available at https://doi.org/10.6084/m9.figshare.c.7869659.v1. The previously published genome sequence for strain ICC168 used in this work is available under accession code FN543502 [https://www.ncbi.nlm.nih.gov/nuccore/FN543502] .The whole genome reference sequence used for ICC8001 is the closely related ATCC43186-derivitive KPPR1 CP009208.1 (https://www.ncbi.nlm.nih.gov/nuccore/CP009208.1). The data that support this

study are available in the supplementary figures, tables and Source Data file

Research involving human participants, their data, or biological material

Policy information about studies with human participants or human data. See also policy information about sex, gender (identity/presentation), and sexual orientation and race, ethnicity and racism.

Reporting on sex and gender	No human participants, data, or biological materials are included in this study
Reporting on race, ethnicity, or other socially relevant groupings	*Please specify the socially constructed or socially relevant categorization variable(s) used in your manuscript and explain why they were used. Please note that such variables should not be used as proxies for other socially constructed/relevant variables (for example, race or ethnicity should not be used as a proxy for socioeconomic status).* *Provide clear definitions of the relevant terms used, how they were provided (by the participants/respondents, the researchers, or third parties), and the method(s) used to classify people into the different categories (e.g. self-report, census or administrative data, social media data, etc.)* *Please provide details about how you controlled for confounding variables in your analyses.*
Population characteristics	*Describe the covariate-relevant population characteristics of the human research participants (e.g. age, genotypic information, past and current diagnosis and treatment categories). If you filled out the behavioural & social sciences study design questions and have nothing to add here, write "See above."*
Recruitment	*Describe how participants were recruited. Outline any potential self-selection bias or other biases that may be present and how these are likely to impact results.*
Ethics oversight	*Identify the organization(s) that approved the study protocol.*

Note that full information on the approval of the study protocol must also be provided in the manuscript.

Field-specific reporting

Please select the one below that is the best fit for your research. If you are not sure, read the appropriate sections before making your selection.

☒ Life sciences ☐ Behavioural & social sciences ☐ Ecological, evolutionary & environmental sciences

For a reference copy of the document with all sections, see nature.com/documents/nr-reporting-summary-flat.pdf

Life sciences study design

All studies must disclose on these points even when the disclosure is negative.

Sample size	Sample size was not determined except for in vivo experiments, where power calculations (using Gpower 3.1) were used to estimate sample size.
Data exclusions	No data were excluded.
Replication	All experiments were replicated independently at least two times.
Randomization	For each animal experiment, mice were randomly assigned to experimental groups by the technicians in the animal facility.
Blinding	Investigators were blinded during group allocation, but were not blinded during the experimental procedure or during the analysis. Blinding is not necessary as the measurements are objective (low to no risk of subjective bias); moreover blinding would difficult monitoring of adverse effects to the infection.

Reporting for specific materials, systems and methods

We require information from authors about some types of materials, experimental systems and methods used in many studies. Here, indicate whether each material, system or method listed is relevant to your study. If you are not sure if a list item applies to your research, read the appropriate section before selecting a response.

Materials & experimental systems

n/a	Involved in the study
☐	☒ Antibodies
☐	☒ Eukaryotic cell lines
☒	☐ Palaeontology and archaeology
☐	☒ Animals and other organisms
☒	☐ Clinical data
☒	☐ Dual use research of concern
☒	☐ Plants

Methods

n/a	Involved in the study
☒	☐ ChIP-seq
☐	☒ Flow cytometry
☒	☐ MRI-based neuroimaging

Antibodies

Antibodies used	Please see Supplementary Data 3.
Validation	Most antibodies used are commercial and have already been validated by the manufacturer using target-deficient cells and/or purified antigen. Anti-KP O1 mAb (C13) was validated in PMID: 1872526 and anti-Citrobacter rodentium was described in PMID: 28146477.

Eukaryotic cell lines

Policy information about cell lines and Sex and Gender in Research

Cell line source(s)	3T3-Swiss albino (CCL-92) is a fibroblast cell that was isolated from disaggregated Swiss mouse embryos in 1962 and obtained from the ATCC.
Authentication	Authenticated by ATCC
Mycoplasma contamination	All cell lines tested negative for mycoplasma.
Commonly misidentified lines (See ICLAC register)	No commonly misidentified cell lines were used in this study.

Animals and other research organisms

Policy information about studies involving animals; ARRIVE guidelines recommended for reporting animal research, and Sex and Gender in Research

Laboratory animals	Female CD-1 and C57BL/6 mice were purchased at 29-31 g weight (5-7 weeks; CD-1 mice) and 18-20g weight (6-8 weeks; C57BL/6 mice) from Charles River UK.
Wild animals	No wild animals were used in this study
Reporting on sex	Female CD-1 and C57BL/6 mice were used in this study; no male mice were used.
Field-collected samples	The study did not involve samples obtained from the field.
Ethics oversight	Work was approved locally by the institutional ethics committee at Imperial College (AWERB).

Note that full information on the approval of the study protocol must also be provided in the manuscript.

Plants

Seed stocks	No plant material was used in this study.
Novel plant genotypes	*Describe the methods by which all novel plant genotypes were produced. This includes those generated by transgenic approaches, gene editing, chemical/radiation-based mutagenesis and hybridization. For transgenic lines, describe the transformation method, the number of independent lines analyzed and the generation upon which experiments were performed. For gene-edited lines, describe the editor used, the endogenous sequence targeted for editing, the targeting guide RNA sequence (if applicable) and how the editor was applied.*
Authentication	*Describe any authentication procedures for each seed stock used or novel genotype generated. Describe any experiments used to assess the effect of a mutation and, where applicable, how potential secondary effects (e.g. second site T-DNA insertions, mosiacism, off-target gene editing) were examined.*

Flow Cytometry

Plots

Confirm that:

- [x] The axis labels state the marker and fluorochrome used (e.g. CD4-FITC).
- [x] The axis scales are clearly visible. Include numbers along axes only for bottom left plot of group (a 'group' is an analysis of identical markers).
- [x] All plots are contour plots with outliers or pseudocolor plots.
- [x] A numerical value for number of cells or percentage (with statistics) is provided.

Methodology

Sample preparation	Bacteria were grown overnight in the indicated growth media, blocked in 5% BSA/PBS for 1 h and stained overnight with primary antibodies or polyclonal anti-sera in at 4°C.<br>Mouse intestinal epithelial cells (IECs) were isolated from 2-cm distal colonic tissue samples, washed in HBSS and incubated at 37°C, 200 rpm for 45 min in IEC dissociation buffer (1× HBSS, 10 mM HEPES, 1 mM EDTA, and 5 µL/mL 2-β-mercaptoethanol). The remaining tissue was removed, and detached cells were collected by centrifugation (2100 xg for 10 min), followed by two DPBS washes at 4°C. Cells were then incubated 5 mins at RT with 50 µg/mL DNase I and passed through a 70-µm cell strainer on ice to ensure a single-cell suspension, pooling samples from the same group and rinsing with 20 mL of cold DPBS. Cell samples enriched in IECs were then blocked in fluorescence-activated cell sorter (FACS) buffer (5% FBS, 2mM EDTA in DPBS) supplemented with Fc block for 10 min and stained for 30 mins at 4°C with primary antibody (anti-C.rodentium) in FACS buffer.<br>Fluorophore-conjugated antibodies were added after 2 washes and incubation was for 1 h (bacteria; in 1%BSA/PBS) or 30 mins (mammalian cells; in FACS buffer). After 2 washes, samples were fixed in 1% PFA/PBS and kept in the dark until flow cytometry analysis.
Instrument	Cytek Amnis CellStream 17-colour, 4-laser system (V-405nm, B-488nm, YG-561nm, R-642nm) flow cytometer (bacteria) and BD LSRFortessa 14 colour analyser with four lasers (Violet 405nm, Blue 488nm, Yellow-Green 561nm and Red 640nm) cell analyser (mouse cells)
Software	Cytek AMNIS CellStream acquisition software 1.3.384 and BD FACSDiva v9.0 Software to collect data. FlowJo v10.10.0 to analyse.
Cell population abundance	Samples were not sorted and so there is no information on post-sort fractions. Flow cytometry was only used for analysis purposes and relevant cell population abundance is provided in figures, supplementary figures, Source Data and/or methods
Gating strategy	Gating strategies including the FSCvsSSC gates are provided in Supplementary Fig 2A (mammalian cells) and Supplementary Fig 2D and 4C (bacterial cells).

- [x] Tick this box to confirm that a figure exemplifying the gating strategy is provided in the Supplementary Information.
